# Supplementary material for: Interhemispheric Pediatric Meningioma, YAP1 Fusion-Positive
Source: Diagnostics (Basel). 2022 Sep 29;12(10):2367. doi: 10.3390/diagnostics12102367 (PMC9601199; doi:10.3390/diagnostics12102367)
Supplement: Supplementary file 1 [file diagnostics-12-02367-s001.zip › Table S1.pdf]

Methylation class	Calibrated Scores for sample 204958100051_R05C01
MNG_BEN_3	0,95438853
MNG_MAL	0,01364493
MNG_BEN_1	0,00856184
MNG_BEN_2	0,00470437
MNG_INT_A	0,00136722
EPN_PFA_1B	0,00076064
NFIB_PLEX	0,00055358
CNS_SARC_CIC	0,00054427
CHORDM	0,00053931
SFT_HMPC	0,00045365
EPN_PFA_1A	0,00041899
MB_G34_III	0,00038440
DIG_DIA	0,00035187
MPNST_TYP	0,00035042
MB_G34_VIII	0,00034438
EWS	0,00032260
EPN_ST_ZFTA_RELA_A	0,00026946
EPN_PFA_1D	0,00026816
HMB	0,00025791
NB_MYCN	0,00022298
MB_G34_VI	0,00022287
MB_G34_V	0,00021393
MELN	0,00020259
MB_SHH_2	0,00020234
ARMS	0,00019973
MB_G34_VII	0,00019968
EPN_PF_SE	0,00019377
MB_G34_II	0,00018639
MB_G34_IV	0,00017481
MB_SHH_1	0,00016498
pedHGG_MYCN	0,00016459
EPN_PFA_2B	0,00015905
PITUI	0,00014533
EPN_MPE	0,00014314
EPN_PFA_1C	0,00014271
EPN_PFA_2A	0,00014023
CPH_PAP	0,00013940
ETMR_C19MC	0,00013915
ATRT_MYC	0,00013631
A_IDH_LG	0,00013568
CPH_ADM	0,00012867
SEGA	0,00012641
NB_TMM_NEG	0,00012567
MB_SHH_3	0,00012357
EPN_PFB_1	0,00012251
ATRT_SHH	0,00011512
MB_G34_I	0,00011392
GCT_TERA	0,00011372
GBM_MES_TYP	0,00011349

ONB	0,00011099
PA_MID	0,00010914
PA_INF	0,00010664
PITAD_GON	0,00010555
NB_TMM_POS	0,00010547
MB_SHH_4	0,00010070
SCHW	0,00010048
CTRL_ADENOPIT	0,00009932
RB	0,00009792
PXA	0,00009689
IO_MEPL	0,00009497
EPN_SPINE	0,00009415
PITAD_ACTH	0,00009399
DHG_G34	0,00009228
DMG_K27	0,00008930
EPN_PFA_2C	0,00008831
PGNT	0,00008435
MB_WNT	0,00008372
CRINET	0,00008193
CPP_AD	0,00008114
pedHGG_RTK2A	0,00007972
ET_PLAG	0,00007791
MB_SHH_IDH	0,00007777
CAUDEQU_NET	0,00007769
DNET	0,00007690
PTPR_B	0,00007578
CNS_NB_FOXR2	0,00007569
MET_MEL	0,00007558
GCT_YOLKSAC	0,00007518
PITAD_PRL	0,00007469
EPN_ST_ZFTA_FUS_C	0,00007334
EPN_PFA_1E	0,00007159
INFLAM_ENV	0,00007131
SNUC_IDH2	0,00007104
CTRL_HEMI	0,00007005
CTRL_REACTIVE	0,00006939
EPN_PFA_1F	0,00006917
CNS_BCOR_ITD	0,00006773
EPN_ST_ZFTA_FUS_E	0,00006739
EPN_YAP	0,00006574
GBM_MES_ATYP	0,00006442
EPN_ST_SE	0,00006415
PIN_RB	0,00006326
A_IDH_HG	0,00006145
pedHGG_RTK1B	0,00006093
MYXGNT	0,00006078
PB_GRP1A	0,00006031
IHG	0,00006008
PLNTY	0,00005753
pedHGG_RTK1A	0,00005711

CN	0,00005708
MNG_INT_B	0,00005549
ERMS	0,00005455
GG	0,00005447
EPN_PFB_2	0,00005310
PITAD_STH_DENSE2	0,00005293
PA_CORT	0,00005210
RMS_MYOD1	0,00005201
MNG_SMARCE1	0,00005175
DGONC	0,00005159
CNS_SARC_DICER	0,00005137
OLIGOSARC_IDH	0,00005067
ABM_MN1	0,00005014
GCT_GERM_A	0,00004934
EPN_PFB_3	0,00004871
EPN_SPINE_SE_A	0,00004838
CHGL	0,00004794
PITAD_STH_SPARSE	0,00004770
MMNST	0,00004730
HGAP	0,00004666
GCT_GERM_KIT	0,00004631
O_IDH	0,00004593
PIN_CYT	0,00004588
LIPN	0,00004475
CTRL_CORPCAL	0,00004421
GBM_RTK2	0,00004401
CPC_AD	0,00004399
GBM_RTK1	0,00004370
ET_BRD4_LEUTX	0,00004070
MPNST_ATYP	0,00004048
RGNT	0,00003992
EPN_PFB_4	0,00003948
CPC_PED	0,00003925
EPN_ST_ZFTA_FUS_D	0,00003849
ETMR_Atyp	0,00003757
LGG_MYB_C	0,00003503
PLASMACYT	0,00003456
PITAD_TSH	0,00003372
AG_MYB	0,00003362
CNS_SCHW_VGLL	0,00003346
DLBCL	0,00003309
NET_PATZ1	0,00003265
PB_FOXR2	0,00003232
CPP_PED	0,00003135
EPN_SPINE_MYCN	0,00002850
PPTID_A	0,00002793
DMG_EGFR	0,00002726
RB_MYCN	0,00002685
NET_PLAGL1_FUS	0,00002675
GNT_A	0,00002654

pedHGG_A	0,00002576
ANTCON	0,00002562
NET_CXXC5	0,00002497
DLGNT_1	0,00002417
HGG_E	0,00002289
EPN_PFB_5	0,00002269
CTRL_HYPOTHAL	0,00002254
EPN_ST_ZFTA_RELA_B	0,00002232
HGG_F	0,00002173
LGG_MYB_B	0,00002136
CTRL_CBM	0,00002107
pedHGG_B	0,00002098
PTPR_A	0,00002080
CTRL_BLOOD	0,00002048
LGG_MYB_D	0,00002014
HGG_B	0,00002014
GBM_PNC	0,00001982
PB_GRP1B	0,00001935
MB_MYO	0,00001925
CTRL_PONS	0,00001917
PPTID_B	0,00001789
EVNCYT	0,00001738
PB_GRP2	0,00001650
pedHGG_RTK1C	0,00001632
EPN_SPINE_SE_B	0,00001606
GBM_CBM	0,00001606
PITAD_STH_DENSE1	0,00001482
PA_INF_FGFR	0,00001478
CTRL_OPTIC	0,00001410
CNS_BCOR_FUS	0,00001387
DLGNT_2	0,00001049
LCH	0,00000969
pedHGG_RTK2B	0,00000811
CTRL_PIN	0,00000704
ATRT_TYR	0,00000000
